# Analysis of the tryptic search space in UniProt databases

**DOI:** 10.1002/pmic.201400227

**Published:** 2014-12-03

**Authors:** Emanuele Alpi, Johannes Griss, Alan Wilter Sousa da Silva, Benoit Bely, Ricardo Antunes, Hermann Zellner, Daniel Ríos, Claire O'Donovan, Juan Antonio Vizcaíno, Maria J Martin

**Affiliations:** European Molecular Biology Laboratory, European Bioinformatics Institute (EMBL-EBI), Wellcome Trust Genome CampusHinxton, Cambridge, UK

**Keywords:** Bioinformatics, Protein isoforms, Sequence redundancy, Trypsin digestion, Variation

## Abstract

In this article, we provide a comprehensive study of the content of the Universal Protein Resource (UniProt) protein data sets for human and mouse. The tryptic search spaces of the UniProtKB (UniProt knowledgebase) complete proteome sets were compared with other data sets from UniProtKB and with the corresponding International Protein Index, reference sequence, Ensembl, and UniRef100 (where UniRef is UniProt reference clusters) organism-specific data sets. All protein forms annotated in UniProtKB (both the canonical sequences and isoforms) were evaluated in this study. In addition, natural and disease-associated amino acid variants annotated in UniProtKB were included in the evaluation. The peptide unicity was also evaluated for each data set. Furthermore, the peptide information in the UniProtKB data sets was also compared against the available peptide-level identifications in the main MS-based proteomics repositories. Identifying the peptides observed in these repositories is an important resource of information for protein databases as they provide supporting evidence for the existence of otherwise predicted proteins. Likewise, the repositories could use the information available in UniProtKB to direct reprocessing efforts on specific sets of peptides/proteins of interest. In summary, we provide comprehensive information about the different organism-specific sequence data sets available from UniProt, together with the pros and cons for each, in terms of search space for MS-based bottom-up proteomics workflows. The aim of the analysis is to provide a clear view of the tryptic search space of UniProt and other protein databases to enable scientists to select those most appropriate for their purposes.

## 1 Introduction

Most of the current MS-based bottom-up proteomics workflows make use of collections of sequences (either proteins or nucleotides) to match peptide sequences to experimental spectra and then to infer the proteins to which those peptides belong 1. The serine protease trypsin is the most used cleaving agent in these workflows.

The Universal Protein Resource (UniProt, http://www.uniprot.org) 2 is among the most used protein sequence and functional annotation providers. Among the UniProt databases (DBs) are the UniProt knowledgebase (UniProtKB) that acts as the central hub for the collection of functional information on proteins and the UniProt reference clusters (UniRef. 3 that merge closely related sequences based on sequence identity. UniProtKB consists of two sections: UniProtKB/Swiss-Prot, which is manually annotated and reviewed, and UniProtKB/TrEMBL, which is automatically annotated and is unreviewed. In the UniProtKB/Swiss-Prot section, protein isoform and variant information is also provided.

The aim of the analysis reported in this study is to provide a clear view of the tryptic search space of UniProt and other protein data sets to enable scientists to select those most appropriate for their purposes. This is all the more pertinent now since proteomics papers in the public domain are still being produced (as well as evaluated and accepted for publication) using as DB the International Protein Index (IPI) 4, well after it was discontinued (on September 2011), hence omitting any new or updated protein sequences. Since comparing tryptic search spaces from different data sets can assist in pinpointing differences between them and help users to understand the reasons behind those differences; in addition to the UniProtKB data sets, we also considered other popular resources, such as the Ensembl 5 and the National Center for Biotechnology Information (NCBI) Reference Sequence (RefSeq) 6 data sets, for this analysis.

We also included information coming from MS proteomics repositories, which provide a global view of large sets of processed mass spectral data. Specifically, we investigated the three most prominent: the PRIDE 7, PeptideAtlas 8, and the Global Proteome Machine database (GPMDB) 9.

In this article, we focus on sequence collections related issues and we then present the results of comparative analysis of the tryptic search space in these various resources and suggestions for their use, including, for instance, the advice not to *a priori* exclude UniProtKB/Swiss-Prot isoforms nor UniProtKB/TrEMBL sequences from UniProt collections.

## 2 Materials and methods

### 2.1 Protein sequence collection

The species analyzed were *Homo sapiens* and *Mus musculus*, for which UniProtKB complete proteome sets 2 were obtained from ftp.uniprot.org/pub/databases/uniprot/current_release/knowledgebase/proteomes/. The other UniProtKB sequence collections were all obtained using the public UniProt web interface. A summary of the sequence data sets is reported in Table1 and Supporting Information Table1 together with the nomenclature adopted for each concrete protein data set and the relationships among data sets. Additional species were also analyzed but are not discussed in any detail in the main text. Information is reported in the Supporting Information Notes, for example, as in Supporting Information Table2.

**Table 1:** Details of the protein data sets used in this study

DB	Species	Release	Set specification	Abbreviation
UniProtKB	*H. sapiens*	2012_10	UniProtKB/Swiss-Prot sequences (canonical and isoforms)	SPI
	*M. musculus*			
UniProtKB	*H. sapiens*	2012_10	UniProtKB/Swiss-Prot sequences (canonical and isoforms) disease-related variant expanded	SPID
UniProtKB	*H. sapiens*	2012_10	UniProtKB/Swiss-Prot sequences (canonical only, without isoforms)	SP
	*M. musculus*			
UniProtKB	*H. sapiens*	2012_10	UniProtKB/Swiss-Prot sequences (canonical and isoforms) variant expanded	SPIV
	*M. musculus*			
UniProtKB	*H. sapiens*	2012_10	UniProtKB complete proteome set sequences (UniProtKB/Swiss-Prot canonical and isoforms plus UniProtKB/TrEMBL, all with KW-0181). The keyword KW-0181 refers to complete proteomes (http://www.uniprot.org/docs/keywlist)	CPI
	*M. musculus*			
UniProtKB	*H. sapiens*	2012_10	UniProtKB complete proteome set sequences (UniProtKB/Swiss-Prot canonical and isoforms plus UniProtKB/TrEMBL, all with KW-0181) disease-related variant expanded	CPID
UniProtKB	*H. sapiens*	2012_10	UniProtKB complete proteome set sequences (UniProtKB/Swiss-Prot canonical only, without isoforms plus UniProtKB/TrEMBL, all with KW-0181)	CP
	*M. musculus*			
UniProtKB	*H. sapiens*	2012_10	UniProtKB complete proteome set sequences (UniProtKB/Swiss-Prot canonical and isoforms plus UniProtKB/TrEMBL, all with KW-0181) variant expanded	CPIV
	*M. musculus*			
UniProtKB	*H. sapiens*	2012_10	UniProtKB whole (UniProtKB/Swiss-Prot canonical and isoforms plus UniProtKB/TrEMBL sequences); this DB is equivalent to the merging of SPI + TR	UPI
	*M. musculus*			
UniProtKB	*H. sapiens*	2012_10	UniProtKB whole (UniProtKB/Swiss-Prot canonical and isoforms plus UniProtKB/TrEMBL sequences) disease-related variant expanded; this DB is equivalent to the merging of SPID + TR	UPID
UniProtKB	*H. sapiens*	2012_10	UniProtKB whole (UniProtKB/Swiss-Prot canonical only, without isoforms plus UniProtKB/TrEMBL sequences); this DB is equivalent to the merging of SP + TR	UP
	*M. musculus*			
UniProtKB	*H. sapiens*	2012_10	UniProtKB whole (UniProtKB/Swiss-Prot canonical and isoforms plus UniProtKB/TrEMBL sequences) variant expanded; this DB is equivalent to the merging of SPIV + TR	UPIV
	*M. musculus*			
UniProtKB	*H. sapiens*	2012_10	UniProtKB/TrEMBL sequences	TR
	*M. musculus*			
UniRef100	*H. sapiens*	2012_10	UniRef100 clustered sequences from CPI	CPIR
	*M. musculus*			
UniRef100	*H. sapiens*	2012_10	UniRef100 clustered sequences from CPID	CPIDR
UniRef100	*H. sapiens*	2012_10	UniRef100 clustered sequences from CPIV	CPIVR
	*M. musculus*			
UniRef100	*H. sapiens*	2012_10	UniRef100 sequences from UPI	UPIR
	*M. musculus*			
UniRef100	*H. sapiens*	2012_10	UniRef100 sequences from UPID	UPIDR
UniRef100	*H. sapiens*	2012_10	UniRef100 sequences from UPIV	UPIVR
	*M. musculus*			

DB	Species	Release	DB	Species	Release	DB	Species	Release
RefSeq	*H. sapiens*	55	Ensembl	*H. sapiens*	68	IPI	*H. sapiens*	09/2011
	*M. musculus*			*M. musculus*			*M. musculus*	

The relationships among the UniProtKB data sets are as follows: SP<SPI<UPI<UPIV, SPID<SPIV<UPIV, UPID<UPIV, CP<CPI<UPIV, CPID<CPIV<UPIV, and TR<UP<UPI; each UniRef100 data set is a subset of the corresponding UniProtKB data set. Data sets not coming from UniProtKB are grouped at the bottom of the table.

**Table 2:** Pairwise comparisons of UniProt data sets tryptic search spaces for human and mouse

DB	Peptides	DB	Peptides	DB	Peptides	DB	Peptides	DB	Peptides
SPI	25 972 (4.7)	SPI	0 (0)	SPI	3 (0.00)	SP	3 (0.00)	SPIV	61 477 (0.9)
SP	0 (0)	SPIV	61 830 (1.0)	CPI	73 687 (1.0)	CPI	99 659 (2.0)	CPI	73 331 (0.9)
Com.	596 385 (27.9)	Com.	622 357 (26.9)	Com.	622 354 (26.9)	Com.	596 382 (27.9)	Com.	622 710 (26.9)
CP	0 (0)	CPIR	0 (0)	CPIV	61 474 (0.9)	CPIVR	0 (0)	UPI	85 453 (1.2)
CPI	19 012 (3.2)	CPI	5777 (1.4)	CPI	0 (0)	CPIV	9494 (1.4)	CPI	0 (0)
Com.	677 029 (24.8)	Com.	690 264 (24.4)	Com.	696 041 (24.2)	Com.	748 021 (22.5)	Com.	696 041 (24.2)
UPI	81 566 (1.0)	UPI	16 554 (2.5)	UPI	0 (0)	UP	85 453 (1.2)	UPIR	0 (0)
CPIV	57 587 (0.6)	UP	0 (0)	UPIV	57 587 (0.6)	CPI	16 554 (2.5)	UPI	16 243 (1.5)
Com.	699 928 (24.1)	Com.	764 940 (22.1)	Com.	781 494 (21.6)	Com.	679 487 (24.7)	Com.	765 251 (22.1)
UPIV	143 040 (1.0)	UPIVR	0 (0)	TR	159 137 (1.1)	TR	154 894 (1.0)	SPI	0 (0)
CPI	0 (0)	UPIV	13 859 (1.7)	SPI	181 615 (17.7)	SPIV	239 202 (13.6)	SPID	22 531 (0.4)
Com.	696 041 (24.2)	Com.	825 222 (20.5)	Com.	440 742 (30.7)	Com.	444 985 (30.5)	Com.	622 357 (26.9)
SPID	22 523 (0.4)	CPID	22 520 (0.4)	CPIDR	0 (0)	UPI	85 271 (1.2)	UPI	0 (0)
CPI	73 676 (1.0)	CPI	0 (0)	CPID	9584 (1.4)	CPID	22 338 (0.4)	UPID	22 338 (0.4)
Com.	622 365 (26.9)	Com.	696 041 (24.2)	Com.	708 977 (23.7)	Com.	696 223 (24.2)	Com.	781 494 (21.6)
UPID	107791 (1.1)	UPIDR	0 (0)						
CPI	0 (0)	UPID	13 823 (1.6)						
Com.	696 041 (24.2)	Com.	790 009 (21.4)						
SPI	11 533 (5.2)	SPI	0 (0)	SPI	0 (0)	SP	0 (0)	SPIV	854 (1.0)
SP	0 (0)	SPIV	953 (2.4)	CPI	136 411 (6.3)	CPI	147 944 (6.2)	CPI	136 312 (6.3)
Com.	502 478 (20.1)	Com.	514 011 (19.8)	Com.	514 011 (19.8)	Com.	502 478 (20.1)	Com.	514 110 (19.8)
CP	0 (0)	CPIR	0 (0)	CPIV	854 (1.0)	CPIVR	0 (0)	UPI	58 057 (2.0)
CPI	9222 (4.3)	CPI	2268 (2.9)	CPI	0 (0)	CPIV	3457 (2.5)	CPI	0 (0)
Com.	641 200 (17.1)	Com.	648 154 (19.7)	Com.	650 422 (16.9)	Com.	647 819 (17.0)	Com.	650 422 (16.9)
UPI	57 925 (2.0)	UPI	7998 (3.4)	UPI	0 (0)	UP	58 057 (2.0)	UPIR	0 (0)
CPIV	722 (1.0)	UP	0 (0)	UPIV	722 (0.7)	CPI	7998 (3.4)	UPI	9263 (1.9)
Com.	650 554 (16.9)	Com.	700 481 (15.8)	Com.	708 479 (15.7)	Com.	642 424 (17.1)	Com.	699 216 (15.9)
UPIV	58 779 (2.0)	UPIVR	0 (0)	TR	194 468 (5.0)	TR	194 237 (5.0)		
CPI	0 (0)	UPIV	7691 (1.9)	SPI	170 033 (15.6)	SPIV	170 755 (15.6)		
Com.	650 422 (16.9)	Com.	701 510 (15.8)	Com.	343 978 (21.8)	Com.	344 209 (21.8)		

Each pairwise comparison is delimited by wider spacing after each “Com.” occurrence, and the two data sets (DB) being compared are indicated next to the numbers of peptides unique to each of them. “Peptides” indicate the number of tryptic peptides for each of the three compartments of the comparisons (I, II, and III in Supporting Information Fig. 2). “Com.” indicates the number of tryptic peptides shared by both data sets in each pairwise comparison. Corresponding percentages of peptides that are found in MS proteomics repositories are reported in brackets for each of the three compartments of the comparisons. Mouse comparisons are highlighted with a light gray background.

The organism-specific UniRef100 files were created using the customized in-house CD-HIT algorithm, which is part of the UniRef pipeline 3. Files containing sequences with variants were generated using the “varsplic.pl” script 10,11 (Supporting Information Notes). A modified variant expansion was also devised in order to limit the expansion to the human variants marked as disease-related in the UniProt human polymorphisms and disease mutations file (http://www.uniprot.org/docs/humsavar, the “humsavar” file), a file concerning all human variants annotated in UniProtKB/Swiss-Prot, including information on disease association and disease name.

Ensembl 5 version 68 data sets were retrieved from ftp.ensembl.org/pub/current_fasta/. RefSeq 6 version 55 data sets were retrieved from ftp.ncbi.nih.gov/refseq/ or the NCBI taxonomy DB. IPI data sets were obtained from ftp.ebi.ac.uk/pub/databases/IPI/current/ and were the last versions produced on September 2011. The retrieval from these resources was done at the same time as the studied UniProt release.

### 2.2 MS-based proteomics repositories collection

PRIDE-identified peptides were downloaded from the PRIDE BioMart (http://www.ebi.ac.uk/pride/prideMart.do) and were filtered to retain only the peptides from human and mouse that were identified in at least five PRIDE experiments to compensate for the data heterogeneity present in PRIDE (following the same approach used in 12). Numbers given for PRIDE human and mouse content (as those reported in Tables2, 3 and 4) are based on these filtered results. The PRIDE content unfiltered numbers (considering peptides that were also identified in less than five experiments) for human and mouse can be found in Supporting Information Fig. 1 and Supporting Information Table3.

**Table 3:** Pairwise comparisons of UniProt CPI data sets tryptic search spaces versus other data sets

Organism	DB	Peptides	DB	Peptides	DB	Peptides
*H. sapiens*	Ensembl	3638 (5.6)	IPI	78 490 (1.1)	RefSeq	9023 (1.7)
	CPI	19 479 (4.2)	CPI	15 437 (0.9)	CPI	95 201 (1.7)
	Com.	676 562 (24.7)	Com.	680 604 (24.7)	Com.	600 840 (27.7)
*M. musculus*	Ensembl	3072 (7.6)	IPI	53 994 (2.5)	RefSeq	19 927 (1.4)
	CPI	13 140 (4.5)	CPI	6943 (1.7)	CPI	51 481 (3.3)
	Com.	637 282 (17.2)	Com.	643 479 (17.1)	Com.	598 941 (18.1)

Each pairwise comparison is delimited by wider spacing after each “Com.” occurrence, and the corresponding two data sets (DB) are indicated next to the numbers of peptides unique to each of them. “Peptides” indicate the number of tryptic peptides for each of the three compartments of the comparisons (I, II, and III in Supporting Information Fig. 2). “Com.” indicates the number of tryptic peptides shared by both data sets in each pairwise comparison. Corresponding percentages of peptides that are found in MS proteomics repositories are reported in brackets for each of the three compartments of the comparisons.

**Table 4:** Pairwise comparisons of human and mouse UniProt UPI data sets tryptic search spaces versus other data sets

Organism	DB	Peptides	DB	Peptides	DB	Peptides
*H. sapiens*	Ensembl	2605 (3.0)	IPI	31 888 (0.8)	RefSeq	6823 (0.8)
	UPI	103 899 (1.7)	UPI	54 288 (1.1)	UPI	178 454 (1.5)
	Com.	677 595 (24.7)	Com.	727 206 (23.2)	Com.	603 040 (27.6)
*M. musculus*	Ensembl	2196 (5.9)	IPI	19 000 (1.5)	RefSeq	16 268 (1.0)
	UPI	70 321 (2.4)	UPI	30 006 (0.8)	UPI	105 879 (2.6)
	Com.	638 158 (17.2)	Com.	678 473 (16.4)	Com.	602 600 (18.0)

Each pairwise comparison is delimited by wider spacing after each “Com.” occurrence, and the corresponding two data sets (DB) are indicated next to the numbers of peptides unique to each of them. “Peptides” indicate the number of tryptic peptides for each of the three compartments of the comparisons (I, II, and III in Supporting Information Fig. 2). “Com.” indicates the number of tryptic peptides shared by both data sets in each pairwise comparison. Corresponding percentages of peptides that are found in MS proteomics repositories are reported in brackets for each of the three compartments of the comparisons.

PeptideAtlas-identified peptides and GPMDB proteotypic peptides were obtained from http://www.peptideatlas.org/builds/ and ftp.thegpm.org/projects/xhunter/libs/eukaryotes/peptide/, respectively. Data from these three repositories was used as originally provided. The retrieval from the three resources was done at the same time as the studied UniProt release (see Table1). The numbers reported in the tables as valuable evidence from the MS proteomics repositories are referred to the presence of one specific peptide in at least one of the repositories. Therefore, unless noted otherwise, they are not referred to the concurrent presence of those specific peptides in the three repositories.

### 2.3 DB pairwise comparisons

After the initial *in silico* tryptic digestion of the protein data sets, only those with six or more amino acids (AAs) were considered for the analysis, as shorter peptides are rarely detected in MS bottom-up proteomics pipelines and lack sequence-specific information 13. Tryptic peptide pairwise comparisons were performed in a similar way to what has been previously described 12: full tryptic cleavage, no missed cleavages, and no initiator methionine cleavage. Details on nontryptic and missed cleavage containing peptides are given in the Supporting Information Notes.

These comparisons split all the tryptic peptides coming from the two data sets being compared (generally DB1 and DB2) into three categories (denoted as I, II, and III in Supporting Information Fig. 2): unique to DB1, shared by both data sets, and unique to DB2. These three lists for each pairwise comparison were used as input to query the MS proteomics repositories.

Upon *in silico* digestion, the sequences of the tryptic peptides corresponding to each entry in the protein data set were recorded, together with their DB accession number and monoisotopic mass 14 (see Supporting Information Notes for details concerning ambiguous and nonstandard residues). By comparing the DB accession numbers corresponding to the three groups of peptides coming from the comparison of two protein data sets (indicated as I, II and III in Supporting Information Fig. 2), it is also possible to identify the accession numbers from DB1, which do not have a sequence representative in DB2, thus changing the focus from peptide sequence to DB accession numbers. As can be seen in Supporting Information Fig. 2, only filtered peptides coming from the *in silico* digestion of the protein data sets can get a match to the peptides coming from repositories: the criterion followed is to have 100% exact sequence match for the entire length of each peptide.

## 3 Results and discussion

In this study, the tryptic search space of the different UniProt sets was compared (Table2). In addition, UniProt complete proteomes data sets were also compared with IPI, RefSeq, and Ensembl (Tables3 and 4).

By using UniProtKB sequence data sets containing canonical plus isoform sequences, or only canonical sequences, it was possible to verify the amount of extra information provided by isoforms and to associate it with the current evidence available in MS proteomics repositories. The same reasoning was applied for variant-expanded data sets and for sequence clustering. Human and mouse were the main focus of the study for these expanded data sets since there is not enough information for the other organisms in UniProtKB for protein isoforms and variant content (Supporting Information Table4).

In the context of protein inference 15, a unique peptide is a peptide that can be unambiguously assigned to a single protein sequence or a group of proteins coming from the same gene (although this second possibility was not explored here). Hence, peptide uniqueness is dependent on the collection of protein sequences considered. These topics, together with exact sequence redundancy (which makes two proteins indistinguishable by MS approaches), underline the importance of having a complete and clear view on the information provided by different data sets.

### 3.1 Sequence redundancy removal

Sequence redundancy does not help in the identification of a protein, since more peptide–protein mapping ambiguity will occur summing to the MS-inherent identification ambiguities 16. Nevertheless, when it is not limited to exact entire entries, sequence redundancy removal from protein data sets eliminates parts of sequences that can produce peptides upon cleavage, which also hinders identifications. In order to explore the effect of the removal of sequence redundancy, we show in detail the effect of sequence clustering on the UniProtKB sequences. In the tables, data were reported for the UniRef100 clustering of the human and mouse CPI, CPID (only human), CPIV, UPI, UPID (only human), and UPIV data sets (Table1 for details and abbreviations).

Sequence clustering of the human UniProt UPI data set removed 41 458 (28%) sequences (corresponding to the UPI vs. UPIR data sets in Supporting Information Table1). In terms of tryptic peptides, UPIR had 16 243 peptides less than UPI (Table5). Accordingly, peptide unicity was around 33 and 37% for UPI and UPIR, respectively (Table5). A total of 15 125 peptides of the 16 243 ones were uniquely produced from single UPI sequences, 5412 from the N-terminal ends, and 7099 from the C-terminal ends. Table2 (UPI/UPIR comparison) shows that the evidence in MS proteomics repositories for the 16 243 lost peptides is low. Similar trends were found for the mouse UPI/UPIR comparison and all the other UniProt human and mouse data sets where redundancy was removed: for example, the comparisons CPI/CPIR, CPID/CPIDR (only human), CPIV/CPIVR, UPID/UPIDR (only human), and UPIV/UPIVR (Tables2 and 5; Supporting Information Tables1 and 5).

**Table 5:** Peptide unicity table for the other UniProt human and mouse data sets

	CPID	CP	CPIV	CPIR	CPIDR	CPIVR	SPI	SPID	SP
*H. sapiens*	718 561 (33.5)	677 029 (52.5)	757 515 (21.6)	690 264 (40.6)	708 977 (40.8)	748 021 (23.0)	622 357 (53.5)	644 888 (47.5)	596 385 (96.5)
*M. musculus*		641 200 (62.2)	651 276 (49.6)	648 154 (53.5)		647 819 (53.5)	514 011 (67.7)		502 478 (97.6)
	SPIV	UPI	UPID	UP	UPIV	UPIR	UPIDR	UPIVR	TR
*H. sapiens*	684 187 (19.8)	781 494 (33.0)	803 832 (32.2)	764 940 (39.5)	839 081 (24.8)	765 251 (37.0)	790 009 (35.8)	825 222 (25.9)	599 879 (44.5)
*M. musculus*	514 964 (66.2)	708 479 (33.0)		700 481 (39.3)	709 201 (32.8)	699 216 (42.0)		701 510 (41.6)	538 446 (46.9)

For each organism and each data set, the total number of tryptic peptides is reported together with the percentage of unique peptides in brackets.

In the comparisons between human and mouse data, for example, the UniProtKB protein sets (either with and without sequence redundancy) and the corresponding data sets from other providers (RefSeq, Ensembl, and IPI; Supporting Information Table 6), the number of peptides unique to the non-UniProtKB data sets always increased after redundancy removal, together with a corresponding increase of the evidence in MS proteomics repositories (which is in general low for these peptides). This indicates a loss of sequence information in UniProtKB upon sequence redundancy removal.

The issue of peptides, resulting from protein cleavage being lost during the clustering redundancy removal, is not limited to UniRef100. The reason behind is exemplified for trypsin cleavage in Supporting Information Fig. 3, where sequence A is merged with sequence B during clustering. This process leads to the loss of the peptide indicated in gray. If sequence A consisted of two distinct sequences (divided at the gap), these two sequences would still be merged with sequence B and the two peptides lost would be the gray ones located at the extremities (one nontryptic and the other tryptic in this example). These losses can occur in any part of the sequence, not only in the central portion as schematized in the figure. Even though search engines have the option to specify the cleaving details (e.g., the specificity) of the proteolytic agent, a question remains whether these type of lost peptides have been properly addressed in the reprocessing efforts performed by MS proteomics repositories (as shown in the repository evidence in Table2 while comparing UniRef100 data sets with the corresponding nonclustered ones). DB comparisons help to quickly fish out these lost peptides. This information, together with peptide unicity, would provide the list of peptides in which to focus on during reprocessing efforts.

There are very few supporting evidences in the MS repositories for these peptides and the reason could simply be that not many searches have been done to track them down. Therefore, it seems advisable to search spectral data sets to check for strong matches against these peptides before deciding to remove them from protein sequence collections.

### 3.2 Variant expansion

Next, natural variants for human and mouse were added into the DB comparisons. Human variation information was taken into account both in its entirety in UniProtKB and as a subset containing only the disease-related variants as explained in Materials and methods. This was done also to reduce sequence redundancy with respect to the expansions produced with all variations. In UniProt release 2012_10, there were 1871 UniProtKB/Swiss-Prot human entries (15.1% of the entries in humsavar) that were directly linked to disease. These entries carried 22 743 distinct feature IDs (34.2% of the total feature IDs in humsavar). Of a total of 67 102 variant entries in humsavar, 338 (0.5%) were associated with I/L variations that are difficult to target with standard proteomics MS approaches, and 50 of those 338 were associated to disease.

Disease-related variant expansion for human, created 25 531 additional tryptic peptides (SPI vs. SPID data sets in Table5) from the 48 116 additional sequences (SPI vs. SPID data sets in Supporting Information Table1). Of those 48 116, 23 743 of them (49%) were created in the canonical sequences (1871 distinct ones), whereas 24 373 (51%) in the isoforms (851 distinct corresponding canonical sequences). The evidence coming from MS proteomics repositories can be observed in Table2 (SPI/SPID comparison). The corresponding numbers for the normal expansion (not limited to disease-related) are reported below.

Regarding the coincidences that might occur between UniProtKB/TrEMBL human tryptic peptides and the additional UniProtKB/Swiss-Prot tryptic peptides generated by variant expansion, it is noteworthy that 4243 UniProtKB/Swiss-Prot peptides generated from the variant expansion had the same sequence than an identical number of UniProtKB/TrEMBL tryptic peptides. Considering that UniProtKB/Swiss-Prot variant expansion produces 61 830 additional tryptic peptides (SPI vs. SPIV data sets in Table5), this corresponded to 6.8% of the additional peptides coinciding. This percentage went down to the range of 0.5–0.7% if the total amount of peptides produced from human UniProt data sets (SP, SPI, SPIV, TR, UPI, and UPIV) were considered.

These 61 830 additional tryptic peptides (SPI vs. SPIV data sets in Table5) came from 126 852 additional sequences (SPI vs. SPIV data sets in Supporting Information Table1). Of those, 66 103 were from UniProtKB/Swiss-Prot canonical sequences (12 437 distinct ones), whereas 60 749 were from UniProtKB/Swiss-Prot isoforms (5321 distinct corresponding canonical sequences). The evidence coming from MS proteomics repositories can be observed in Table2 (SPI/SPIV comparison).

From the UniProtKB/Swiss-Prot perspective, in addition to the 61 830 human additional peptides created by the variant expansion, there were 770 additional peptides that by chance coincided with peptides from the SPI DB. Therefore, also the level of peptide coincidence upon variant expansion within UniProtKB/Swiss-Prot was negligible. These peptides could be found by comparing the SPIV DB with an equivalent data set, where all the additional variant-containing sequences had been substituted by their corresponding canonical or isoform ones.

The observed effects in mouse data were different. Due to the substantially lower amount of mouse variation data available (Supporting Information Table4), the effect of UniRef100 clustering on the CPIV data set resulted in a number of entries in the corresponding CPIVR data set (Supporting Information Table1), which was lower than the number of entries in CPIR. As shown before, this trend is the opposite one to human.

With respect to the sequence redundancy introduced by variant expansion, it might not dramatically affect protein grouping in the process of inferring proteins. For instance, in passing from human UPI to UPIV, there is an 86% increase in the number of sequences (from 148 042 to 274 894 see Supporting Information Table1), which corresponds to a 25% decrease in the number of data set unique peptides. This might indicate that the additional sequence redundancy introduced should be mainly found among those entries that are being expanded with variation data.

It is noteworthy here that the disease-related variant expansion human DB UPID had quite less entries than the UPIV database (Supporting Information Table1) and that the sequence unicity of UPID became very similar to the UPI one (Table5). In terms of MS proteomics repository content, only very few variant-containing peptides were found for UPIV and consequently, the same applies for UPID.

A possible limitation of the content of MS proteomics repositories is that "if you don't search for it, you'll never find it." If the data sets used for the searches (by submitters to PRIDE or during the reprocessing for GPMDB and PeptideAtlas) do not contain variation information, it is not possible to find evidence for variant-containing peptides in the repositories.

### 3.3 UniProtKB versus IPI: MS proteomics repositories content

Since IPI has been extensively used by the proteomics community, we report here the comparison between UniProtKB and IPI. Details of the comparisons between IPI, Ensembl, and RefSeq against the UniProtKB complete proteomes can be found in the Supporting Information Notes.

In terms of content of MS proteomics repositories, not many peptides were missing from UniProtKB when compared to the corresponding IPI data sets. From Table4 it can be seen that when comparing IPI to UPI, 3.9% human and 2.6% mouse peptides do not have an equivalent sequence in UniProtKB. In addition, only a very small proportion of these peptides had MS repository evidence, namely 240 (0.7%) and 285 (1.5%) peptides, respectively (see panels A and B in Supporting Information Fig. 1). The evidence was even less for the peptides that are concurrently found in the three repositories (the central intersections of the Venn diagrams in Supporting Information Fig. 1). Panels C and D in Supporting Information Table1 show that, when comparing them to, respectively, panels A and B, the filtering strategy applied to PRIDE (peptides present in at least five different PRIDE experiments) did not significantly affect the results in terms of the peptides concurrently found in the three repositories with respect to the changes in the number of peptides exclusive to PRIDE.

When compared with SPI, the number of peptides unique to IPI increased more than threefold for human (1547 peptides with evidence) and slightly less than ninefold for mouse (9823 with evidence). So, it can be concluded that the highest contribution in terms of IPI coverage comes from the UniProtKB/TrEMBL data sets.

### 3.4 Peptide unicity for the human UniProtKB UPI data set

Among the UniProtKB human protein sets, the unicity of a tryptic peptide within the UPI data set is the most conservative way to evaluate it. Evaluation of the unicity in the variant-expanded data sets, such as UPIV and UPID, would result in an excessive penalization caused by the variant-expanded entries.

Table5 and Supporting Information Table5 show that peptide unicity ranged from 19.8 (human SPIV) to 97.6% (mouse SP) for UniProtKB, and from 33.4 (Ensembl human) to 75.7% (RefSeq mouse) for the other DBs.

In the Supporting Information Notes, details on the human peptides containing the ambiguous residues X, B, and Z and their effect on peptide unicity are reported, together with the related information from MS proteomics repositories.

Excluding the X-, B-, and Z-containing unique peptides, 81 444 UniProtKB human sequences (55.0% of a total of 148 042) were found with at least one unique tryptic peptide. Of those, 19 756 were from UniProtKB/Swiss-Prot (24.3%; 11 153 canonical and 8603 isoforms) and 61 688 from UniProtKB/TrEMBL (75.7%). Of the 81 444 entries, 68 224 (83.8%, 6715 from UniProtKB/Swiss-Prot and 61 509 from UniProtKB/TrEMBL) had a protein existence (PE) value different than 1 (“Evidence at protein level”; for details about PE see http://www.uniprot.org/manual/protein_existence). The highest number of unique peptides per sequence was 246 for the UniProtKB/Swiss-Prot entry Q14204 (4646 AAs), which ranked in position 126 among all the human UniProtKB entries (i.e., the human UPI data set), sorted by decreasing sequence length. The unique tryptic peptide that was repeated many times inside the same sequence was LTMMGTR that was found 27 times in the sequence Q6ZWG8.

After removal of the X-, B-, and Z-containing peptides, the unique peptides left for the human UPI data set were 252 124 of which 6% (15 234) were isoform-specific unique peptides. This highlights the importance of including isoforms in sequence collections. In addition, 55 994 adjunctive unique peptides (thus bringing the total to 308 118) from 4447 UniProtKB/Swiss-Prot entries (2290 of which are not among the 81 444 above mentioned) were found when a peptide was still considered unique if it was found among different isoforms (canonical sequence included) of the same UniProtKB/Swiss-Prot entry. These "entry-specific" unique peptides were not considered further in this numerical analysis, but are key to evaluate gene-level peptide unicity.

In order to use data only from the peptides that are present in the MS proteomics repositories, we observed that the GPMDB human peptides had a length up to 51 AAs, PeptideAtlas up to 66, and PRIDE up to 84 (only seven peptides are longer than 66 AAs and they all contain tryptic missed cleavages). Finally, PRIDE (filtered for five experiments as explained in Materials and methods) contained peptides up to 66. So, we decided to explore on unique peptides of length up to 66 AAs.

The number of tryptic unique peptides (ambiguous sequences excluded, as before) from the UPI data set with a length up to 66 AAs was 248 675. Among these, 30 peptides contained "U" residues (selenocysteine) and only one of these had experimental evidence in PRIDE. In total, 20 848 of these peptides had experimental evidence in at least one of three MS proteomics repositories.

In total, 4197 (1.7%) of the 248 675 peptides concurrently have evidence in all the MS proteomics repositories. They come from 1302 UniProtKB entries (1246 UniProtKB/Swiss-Prot canonical sequences, 10 UniProtKB/Swiss-Prot isoforms, and 46 UniProtKB/TrEMBL sequences). The number of unique peptides per entry ranged from 1 to 131. The PE values for the UniProtKB/Swiss-Prot entries ranged from 1 to 5 (57 entries with PE other than 1) and from 1 to 4 for UniProtKB/TrEMBL entries (41 entries with PE other than 1).

In conclusion, even in this conservative situation (human, which is the best annotated species; *in silico* digestion of the protein data set with only one cleaving agent without missed cleavages; excluding from the digested protein data set entry-specific unique peptides and X-, B-, and Z-containing ambiguous peptides; evaluation of tryptic peptide unicity within the UniProt UPI data set; PRIDE content filtered to five experiments; repository content up to 66 AAs in length and finally concurrent evidence from the three MS proteomics repositories) there is room for enhancement of the PE-value assignment in UniProt. For instance, one UniProtKB/Swiss-Prot entry (O75558) has a PE-value of 2, having nine unique tryptic peptides found in the three MS proteomics repositories. Other two UniProtKB/Swiss-Prot entries (O60361 and Q9H853) have a PE-value of 5 with one unique tryptic peptide found in the three MS proteomics repositories. Finally, twelve UniProtKB/TrEMBL entries (A2NJV5, A8MUW5, D3DTH7, E7EVA3, E9PAU2, E9PGZ2, H0Y4K8, H0Y7A7, H0Y8×4, Q0ZCH6, Q5NV62, and Q5NV86) have a PE-value of 4, having one unique tryptic peptide found in the three MS proteomics repositories.

### 3.5 UniProtKB versus Ensembl, IPI, or RefSeq

When comparing UniProtKB, either complete proteomes (Table3) or other data sets (Table4 and Supporting Information Table 6) to other DBs, some general considerations could be drawn. The MS proteomics repositories content was generally low with respect to the total number of peptides included in the comparisons, for instance, a 27% cumulative maximum of peptides in the comparison between human SP and RefSeq, and 17% between mouse SP and RefSeq.

The amount of extra sequence information provided by the human and mouse isoforms included in UniProtKB/Swiss-Prot and UniProtKB/TrEMBL was evident by looking closely at the results from the pairwise comparisons performed against CPI (or SPI) data sets with those performed against CP (or SP) data sets, or in the comparisons performed against SPI data sets with those performed against the UPI ones. For instance, in the case of human the biggest effect of UniProtKB/Swiss-Prot isoforms provided a 43% decrease in the number of peptides unique to RefSeq when compared to the UniProtKB/Swiss-Prot canonical sequences alone. In the case of mouse, the largest effect of UniProtKB/TrEMBL entries provided a 98% reduction in the number of peptides unique to Ensembl with respect to UniProtKB/Swiss-Prot alone.

In addition, the loss of information upon human sequence clustering can have an effect as big as providing about a fourfold increase in peptides unique to Ensembl, when looking at the results from the comparisons UPI/Ensembl versus UPIR/Ensembl. To summarize, the CPI data sets matched well with the Ensembl data sets (coming directly from the corresponding genomes), but matched increasingly less well with IPI and RefSeq. In addition, the UPI data sets carried more sequence information than the corresponding CPI data sets.

## 4 Concluding remarks

From the analyses performed in this study, these are the main conclusions that can be extracted:

(i) If a maximal sequence coverage (also compared to the previously generated IPI data sets) is sought, then the whole UniProtKB content for the corresponding organisms should be used (UniProtKB UPI data sets).
(ii) If a maximal correspondence with the current underlying Ensembl genome is sought, then UniProtKB complete proteome sets should be used. Indeed, these sequence collections are the proposed substitutes of IPI 12. Table3 shows that the added value of the UniProtKB complete proteomes (CPI data sets) is due to the well-established pipelines between UniProt and Ensembl 2. In fact, the number of peptides unique to Ensembl shown in Table3 is the lowest one, when compared to the peptides unique to RefSeq and IPI, meaning the highest concordance between CPI data sets and Ensembl. Furthermore, UPI data sets (whole UniProt organism-specific content) contain additional peptide-level sequence information compared to the CPI data sets for human and mouse (Table4; see Supporting Information Notes “UniProt complete proteomes and other sequences”).
(iii) If variation data need to be considered in a given study, then a variant-expanded data set is the proper choice. In this case, an additional choice consists in focusing on a subset of variation considering only the ones directly linked to disease, obtaining a data set focusing on detrimental variations with a lower sequence redundancy in the variant-expanded sequence data set. Human variation data have increased as UniProt has developed a pipeline to import high-quality 1000 Genomes 17 and COSMIC 18 nonsynonymous single AA variants from Ensembl variation 19.
(iv) If sequence redundancy is critical in the analysis, then an organism-specific UniRef100 clustered data set is the proper choice bearing in mind that some tryptic peptides are lost during the sequence clustering. Furthermore, organism-specific files similar to the UniRef100 sequence collections are used in the initial steps of the construction of the peptide spectral libraries from the National Institute of Standards and Technology (peptide.nist.gov). In order to address the peptide loss, it would be useful to exploit global mass spectra reprocessing from MS proteomics repositories to check for evidences on the peptides removed during the clustering process. Another way to address sequence redundancy, while not affecting the peptide content, is to remove only those entries that have exactly the same length and sequence.
(v) UniProtKB/Swiss-Prot isoform sequences and the appropriate UniProtKB/TrEMBL sequences are included in the UniProtKB complete proteome sets. The analysis of peptides present in isoforms has shown that it is highly advisable not to discard isoforms.
(vi) The analysis of the UniProtKB/TrEMBL peptide content of the complete proteome sets has shown that it is important to include UniProtKB/TrEMBL sequences in the searches to provide a broader sequence coverage. By not doing so, a large amount of valuable sequence information will be lost (Supporting Information Fig. 4). The lack of manual annotation in UniProtKB/TrEMBL entries is not a valid reason to discard these sequences a priori. This is particularly important for MS matching where, in many cases, missing a sequence in the protein sequence data set might cause missing matches for good spectra.
(vii) The mismatch between the number of tryptic peptides from sequence data sets and the fewer peptides that have evidence in the MS proteomics repositories could be reduced both by considering peptides with missed cleavages sites and peptides obtained with other cleaving rules (trypsin/P and other cleaving agents). Even so, the gap would be addressed in a much more efficient way by regularly performing a global spectral reprocessing against appropriate and updated sequence collections. We strongly recommend the reprocessing of large collections of MS spectra using sequence collections from UniProtKB that contain as much sequence information as possible.
(viii) Integrating the tryptic search space (or a search space from any other cleaving agent) from protein data sets with the peptide-level identifications from the main MS proteomics repositories and the unicity evaluation is a way of adding annotations to the corresponding proteins. One clear example is the use of this information to annotate protein sequences in UniProtKB to assign the appropriate PE-value and to enrich the discovery of sequences of interest and focus curation efforts. Isoform- or variant-specific unique peptides can also be identified for annotation purposes.
(ix) These type of analyses can thus help in deciding what to import into UniProtKB from other data sets with the added value of MS evidence and can detect potential proteotypic/quantotypic peptide candidates to be used in targeted proteomics workflows, such as SRM, including isoforms and sequence variants.

In the near future, UniProt will integrate all the valuable experimental information coming from the MS-driven proteomics data publicly available in MS proteomics repositories.

## Abbreviations

AA: amino acid
DB: database
GPMDB: Global Proteome Machine Database
IPI: International Protein Index
PE: protein existence
UniProt: universal protein resource
UniProtKB: UniProt knowledgebase

## References

[1] Aebersold R, Mann M (2003). Mass spectrometry-based proteomics. Nature.

[2] UniProt Consortium (2013). Update on activities at the Universal Protein Resource (UniProt) in 2013. Nucleic Acids Res.

[3] Suzek BE, Huang H, McGarvey P, Mazumder R, Wu CH (2007). UniRef: comprehensive and non-redundant UniProt reference clusters. Bioinformatics.

[4] Kersey PJ, Duarte J, Williams A, Karavidopoulou Y (2004). The International Protein Index: an integrated database for proteomics experiments. Proteomics.

[5] Flicek P, Ahmed I, Amode MR, Barrell D (2013). Ensembl 2013. Nucleic Acids Res.

[6] Pruitt KD, Tatusova T, Brown GR, Maglott DR (2012). NCBI Reference Sequences (RefSeq): current status, new features and genome annotation policy. Nucleic Acids Res.

[7] Vizcaino JA, Cote RG, Csordas A, Dianes JA (2013). The proteomics identifications (PRIDE) database and associated tools: status in 2013. Nucleic Acids Res.

[8] Deutsch EW (2010). The PeptideAtlas Project. Methods Mol. Biol.

[9] Craig R, Cortens JP, Beavis RC (2004). Open source system for analyzing, validating, and storing protein identification data. J. Proteome Res.

[10] Kersey P, Hermjakob H, Apweiler R (2000). VARSPLIC: alternatively-spliced protein sequences derived from SWISS-PROT and TrEMBL. Bioinformatics.

[11] Hermjakob H, Fleischmann W, Apweiler R (1999). Swissknife—“lazy parsing” of SWISS-PROT entries. Bioinformatics.

[12] Griss J, Martin M, O'Donovan C, Apweiler R (2011). Consequences of the discontinuation of the International Protein Index (IPI) database and its substitution by the UniProtKB “complete proteome” sets. Proteomics.

[13] Cox J, Hubner NC, Mann M (2008). How much peptide sequence information is contained in ion trap tandem mass spectra?. J. Am. Soc. Mass Spectrom.

[14] Haynes WM, Lide DR, Bruno TJ (2012). CRC Handbook of Chemistry and Physics.

[15] Nesvizhskii AI, Aebersold R (2005). Interpretation of shotgun proteomic data: the protein inference problem. Mol. Cell. Proteomics.

[16] Koskinen VR, Emery PA, Creasy DM, Cottrell JS (2011). Hierarchical clustering of shotgun proteomics data. Mol. Cell. Proteomics.

[17] Abecasis GR, Altshuler D, Auton A, Brooks LD (2010). A map of human genome variation from population-scale sequencing. Nature.

[18] Forbes SA, Bindal N, Bamford S, Cole C (2011). COSMIC: mining complete cancer genomes in the catalogue of somatic mutations in cancer. Nucleic Acids Res.

[19] Chen Y, Cunningham F, Rios D, McLaren WM (2010). Ensembl variation resources. BMC Genomics.

