# Supplementary material for: Analysis of the tryptic search space in UniProt databases
Source: Proteomics. 2014 Dec 3;15(1):48–57. doi: 10.1002/pmic.201400227 (PMC4298651; doi:10.1002/pmic.201400227)
Supplement: Supplementary file 1 [file pmic0015-0048-sd1.docx]

**SUPPLEMENTARY MATERIALS**

**Supplementary Notes**

In the following document, details are provided for the following topics.

UniProt sequences not belonging to the complete proteomes 2

Notes on UniProt human data sets 2

Details on the 'varsplic.pl' script 3

Practical example of the 'varsplic.pl' script usage 4

Non-tryptic and missed-cleavages-containing peptides in MS proteomics repositories 5

Ambiguous and non-standard residues in sequence data sets 5

UniProt 2012_10 and MS proteomics repositories non-standard AAs 6

Human UniProt UPI data set unicity contribution from human X-containing peptides 6

Human UniProt UPI data set unicity contribution from human B-containing peptides 6

Human UniProt UPI data set unicity contribution from human Z-containing peptides 7

Human UniProt UPI data set unicity contribution from human peptides concurrently containing X, B and Z residues.. 7

Ambiguous residues in MS proteomics repositories 7

UniProt complete proteomes (CPI data sets) vs. Ensembl 8

UniProt complete proteomes (CPI data sets) vs. IPI 9

UniProt complete proteomes (CPI data sets) vs. RefSeq 10

Legends of the Supplementary Figures 11

Supplementary Figures 12

Supplementary Tables 16

***UniProt sequences not belonging to the complete proteomes***

A complete proteome is defined as the entire set of proteins expressed by a specific organism. The sources of the available different UniProtKB complete proteomes are the available genomes from the International Nucleotide Sequence Database Collaboration (INSDC), Ensembl and Ensembl Genomes. For INSDC, all annotated proteins are imported into UniProtKB (UniProtKB/TrEMBL) but only those proteins coming from complete, annotated genomes and WGS genomes detected as complete will be tagged with the keyword "complete proteome". For Ensembl, all predicted protein sequences are mapped to UniProtKB under stringent conditions: 100% identity over 100% of the length of the two sequences. Any Ensembl sequence found to be absent from UniProtKB is imported. All UniProtKB entries that map to an Ensembl peptide are used to build the proteome. They are tagged and a cross-reference is added. UniProt has also defined a set of "reference proteomes" that are landmarks in the proteome space. Reference proteomes have been selected, among the complete proteomes, to provide a broad coverage of the tree of life, and constitute a representative cross-section of the taxonomic diversity to be found within UniProtKB.

UniProtKB also contains additional sequences with respect to the complete proteome ones as shown in Supplementary Figure 4. Some of the reasons are:

- exact sequence redundancy within UniProtKB/TrEMBL (partial genome assemblies among the causes). This accounts for 14.5% of the 63,148 UniProtKB/TrEMBL human sequences not belonging to the complete proteome. However removing this redundancy increases peptide unicity by only 0.2%.

- some UniProtKB/TrEMBL entries are not considered as new isoforms, or variants, or sequence conflicts or prone to be deleted, until manual curation is performed, thus letting those entries leave UniProtKB/TrEMBL and enter (or be merged into) UniProtKB/Swiss-Prot entries.

For human, the amount of UniProtKB/TrEMBL entries which have the same sequence as a UniProtKB/Swiss-Prot variant-containing sequence, is 613 (around 0.5% of all the UniProtKB/TrEMBL entries). While the number of UniProtKB/TrEMBL entries with an identical sequence to UniProtKB/Swiss-Prot canonical or isoform sequences is 1,457, i.e. 1.3% of all the human UniProtKB/TrEMBL entries.

- six human UniProtKB/Swiss-Prot entries are not part of the human complete proteome since they don't map to the human reference genome. They are the protein accessions P69208, P01858, P01358, P02728, P02729 and P22103. This can be seen in Table 2 for the comparison between SPI and CPI. Similar examples exist, for instance, for *C. elegans*, *Bos taurus*, *A. thaliana* and *D. melanogaster*.

***Notes on UniProt human data sets***

In the human UPI data set there are 139 accessions which do not have any tryptic peptides longer than 6 AAs. The corresponding sequences are 4 to 60 AAs long. Five sequences are from UniProtKB/Swiss-Prot (between 4 and 51 AAs) and 134 sequences are from UniProtKB/TrEMBL (length between 4 and 60 AAs). This indicates that not much is lost if only peptides longer than 6 AAs are considered.

The average length of the tryptic peptide for the human UniProtKB UPI data set is 15.2 AAs. The number changes to 16.2 AAs when only peptides longer than 6 AAs are considered.

The human UniProtKB UPI data set has:

- 148,042 sequences; of those 36,991 are from UniProtKB/Swiss-Prot (25.0%; 20,233 canonical plus 16,758 isoforms) and 111,051 from UniProtKB/TrEMBL (75.0%).

- 781,494 tryptic peptides (6 or more AAs, no missed cleavages); of those 18,207 (2.3% of the total) have 51 or more AAs. This means that the overall contribution from peptides which could be difficult to target *via* standard proteomics MS techniques, is low.

- 257,506 tryptic peptides (33.0% of all the 781,494 ones) are found uniquely in a single sequence among all the 148,042 ones (obviously one sequence can contain more than one unique tryptic peptide: indeed these 257,506 unique tryptic peptides come from 83,901 distinct sequences); 8,788 of these 257,506 peptides (3.4%) have 51 or more AAs. This means that the contribution to unicity from peptides which could be difficult to target *via* standard proteomics MS techniques, is low.

- 47.9% (123,259 peptides) of the 257,506 unique tryptic peptides come from 19,760 distinct UniProtKB/Swiss-Prot (11,155 canonical and 8,605 isoforms) sequences (53.4% of the 36,991 UniProtKB/Swiss-Prot sequences and 13.4% of the 148,042 UniProt sequences); 3,506 of these 123,259 peptides (2.8%) have 51 or more AAs.

- 52.1% (134,247 in number) of the 257,506 unique tryptic peptides come from 64,141 distinct UniProtKB/TrEMBL sequences (57.7% of the 111,051 UniProtKB/TrEMBL sequences and 43.3% of the 148,042 UniProt sequences); 5,282 of these 134,247 peptides (3.9%) have 51 or more AAs.

***Details on the 'varsplic.pl' script***

UniProt collections including variant expansion were created using the publicly available and documented 'varsplic.pl' Perl script (ftp.ebi.ac.uk/pub/software/swissprot/varsplic/). The script works on UniProtKB/Swiss-Prot flat files which can be retrieved from the UniProt FTP and integrated with the appropriate UniProtKB/TrEMBL files (also available from the UniProt FTP) as needed, when generating the fasta files used in this work.

The 'varsplic.pl' script gives access to the following sequences (apart from the canonical ones, www.uniprot.org/faq/30):

- the ones tagged as alternative sequences (VAR_SEQ sequence annotation feature, see www.uniprot.org/manual/var_seq, www.uniprot.org/manual/alternative_products and www.uniprot.org/manual/sequence_annotation). These sequences are also directly available *via* the UniProt website for all the species (ftp.uniprot.org/pub/databases/uniprot/current_release/knowledgebase/complete/uniprot_sprot_varsplic.fasta.gz).

- the ones tagged as natural variants (VARIANT sequence annotation feature, see www.uniprot.org/manual/variant).

- the ones tagged as sequence conflicts (CONFLICT sequence annotation feature, see www.uniprot.org/manual/conflict). These sequences are not involved in this study.

The exact combination of isoform (or canonical) sequence and natural variant that 'varsplic.pl' creates can be recognized from the modified accession number and fasta headers that the Perl script provides.

For a UniProt entry that has "n" alternative products (i.e. canonical sequence plus isoform sequences) and "y" variants, the maximum number of sequences that can be created by 'varsplic.pl' is "n·(y+1)".

This number is the maximum theoretical one. In practical terms the number of produced sequences can be less than that due to the checks that 'varsplic.pl' performs on the UniProt flat file. For instance, if variant-containing regions are missing in some of the isoforms, the corresponding additional sequences are not produced.

Having in mind the schema of the pairwise comparisons (Supplementary Figure 2) and the data in Table 2, in order to retrieve the number of 4,243 UniProtKB/Swiss-Prot peptides generated from the variant expansion that coincide at the sequence level with an identical number of UniProtKB/TrEMBL tryptic peptides, two ways can be followed:

a) perform the comparison between UPI and UPIV and take the peptides uniquely found in UPIV; perform the comparison between SPI and SPIV and take the peptides uniquely found in SPIV; perform a Venn diagram of these two lists and take the non-shared sequences.

b) perform the comparison between SPIV and TR (data not shown) and take the peptides uniquely found in TR; perform the comparison between SPI and TR and take the peptides uniquely found in TR; perform a Venn diagram of these two lists and take the non-shared sequences.

The modified 'varsplic.pl' script that we designed adds the corresponding feature IDs (see main text) in the fasta header of the additional sequences. It is thus straightforward to retrieve only those sequences which contain the list of feature IDs directly linked to disease (some additional steps are required with respect to what detailed below for the unmodified script).

***Practical example of the 'varsplic.pl' script usage***

A practical example of the script usage implies the only pre-requisite of having a Perl programming language package installed (the modified 'varsplic.pl' script works in the same way as the unmodified one):

- download the 'varsplic.pl' script at ftp.ebi.ac.uk/pub/software/swissprot/varsplic/varsplic.pl

- download and decompress the SwissKnife Perl module needed for the script to be able to interpret UniProt flat files from ftp.ebi.ac.uk/pub/software/swissprot/Swissknife/Swissknife_1.70.tar.gz (or any more recent version)

- download and decompress a UniProtKB/Swiss-Prot flat file, like for instance ftp.uniprot.org/pub/databases/uniprot/current_release/knowledgebase/taxonomic_divisions/uniprot_sprot_human.dat.gz

- run the varsplic script against the UniProt flat file with, for instance, these arguments:

perl varsplic.pl -input uniprot_sprot_human.dat -fasta expanded.fasta -which full -count -varseq -variant -showdesc

- download all the human UniProtKB/TrEMBL sequences in fasta format (save this file as trembl.fasta): www.uniprot.org/uniprot/?query=(organism%3a%22Homo+sapiens+%5b9606%5d%22)+AND+reviewed%3ano&force=yes&format=fasta

The 'varsplic.pl' line used will produce an ouput fasta file ("expanded.fasta") which is identical to what we have here called the UniProt human SPIV data set.

The UniProt website filtering used will produce a fasta file (trembl.fasta) which is identical to what we have called the UniProt human TR data set.

Merging these two fasta files will produce the UniProt human UPIV data set. In a Microsoft Windows operating system merging can be done with, for instance, this command line:

copy /b expanded.fasta+trembl.fasta UPIV.fasta

The 'varsplic.pl' script produces additional sequences as described for the switch "full" of the option -which: a new record is generated for every existing sequence in the database (i.e. the canonical sequence), plus one new record for each alternative form (isoforms and variant combinations); new records are produced for all existing records in the input file (i.e. the canonical sequences) as well as for all alternative forms (provided that they pass the checks described above).

Accession numbers for each new entry are constructed as follows:

parental_AC_number -alternative_product_number -variant_number

Such that P12345-00-00 would possess the same sequence as the parent record (i.e. canonical sequence), and P12345-01-02 would possess the splicing variations belonging to the first alternative splice form, the variant features belonging to the second alternative variant form. Entries not affected by variant expansion retain their original accession numbers.

***Non-tryptic and missed-cleavages-containing peptides in MS proteomics repositories***

Even though we used exact tryptic cleavage with no missed-cleavages, we also estimated how many tryptic peptides are reported in the MS proteomics repositories with respect to the non-tryptic ones (which may result for instance from trypsin cleavage at the C-termini of protein sequences or from other cleaving agents). Peptides displaying K or R residues at their C-termini were counted thus showing that the majority of peptides were from tryptic origin (Supplementary Table 3). Among the peptides bearing K or R at their C-terminal extremity, we also estimated the peptides containing one or more tryptic missed-cleavages since these peptides will not match with peptides from sequence data sets due to the exact tryptic cleavage rule that we applied. Sites such as KP or RP were excluded from the missed-cleavage estimation since they are not targeted by the trypsin cleaving rules that we adopted. The results showed that the peptides containing tryptic missed-cleavage sites ranged from about 13% to about 76% and that GPMDB is the most stable repository in terms of percentages of peptides with missed-cleavages with a standard deviation of 2 compared to 14 for PRIDE and 8 for PeptideAtlas (Supplementary Table 3).

From the data available in Table 2, Table 3, Table 4, Supplementary Table 6 and Supplementary Table 3 it can be inferred that, by excluding peptides containing missed-cleavages sites, the numbers of tryptic peptides from the repositories which have a corresponding sequence from the protein data set *in silico* digests range between 43.8% for yeast and 72.5% for *C. elegans*. The reasons for not having higher percentages can be many. Among others: peptide "flyability", peptide lengths (as shown, neither affecting much data sets digests nor MS proteomics repository content) and data set sequence content that has changed over time. The latter reason probably being the most relevant one.

***Ambiguous and non-standard residues in sequence data sets***

Standard atomic weights were used to calculate monoisotopic tryptic peptide masses (residues plus a molecule of water). The "X" (unknown residue; Xaa), "B" (asparagine Asn or aspartic acid Asp; Asx) and "Z" (glutamic acid Glu or glutamine Gln; Glx) residues were not included in mass calculations since they each represent more than one molecule with different molecular weight. However the "J" (leucine Leu or isoleucine Ile; Xle), "O" (pyrrolysine; Pyl, a genome-encoded non-standard aminoacid) and "U" (selenocysteine; Sec, a genome-encoded non-standard aminoacid) residues were included in the calculations.

The NCBI data sets (like the RefSeq ones) contain all the above mentioned AAs. Ensembl data sets do not contain "B", "J", "O" and "Z" residues. IPI data sets do not contain "J", "O" and "U" residues while UniProt data sets do not contain "J" residues. AA statistics for UniProt are reported in Supplementary Table 7.

***UniProt 2012_10 and MS proteomics repositories non-standard AAs***

Regarding the whole UniProtKB sequence content in terms of genome-encoded non-standard AAs O and U:

- 45 sequences (29 UniProtKB/Swiss-Prot canonical and 16 UniProtKB/TrEMBL) bear a single occurrence of the O residue. This turns out in 33 tryptic peptides (23 unique ones) which span a length between 8 and 49 AAs. These 45 sequences all belong to the microbial and bacterial world (none of the species included in this work is represented). The protein existence field is equal to 1 (evidence at the protein level) for 5 of these entries (11.1%). MS proteomics repositories evidence is absent for O-containing peptides.

- 1,780 sequences (250 UniProtKB/Swiss-Prot canonical, 32 UniProtKB/Swiss-Prot isoforms e 1,498 UniProtKB/TrEMBL) bear a total of 1,968 U residues. This turns out in 1,052 tryptic peptides (838 unique ones) which span a length between 6 and 78 AAs and bear between 1 and 7 repetitions of the U residue in their sequence. These 1,780 sequences span a wide range of taxonomies (e.g. from human to bacteria). The species in the paper are all represented except *A. thaliana* and *S. cerevisiae*. The protein existence field is equal to 1 (evidence at the protein level) for 72 of these entries (4.0%). MS proteomics repositories evidence is limited to 5 entries in PRIDE content for *H. sapiens*; only one of these sequences is found among the 1,052 tryptic peptides above cited, namely the sequence KPNSDULGMEEK which is found in the human Q9C0D9 entry (PE=1) and in the *Pongo abelii* Q5NV96 entry (PE=2). It must be noted that in the human PRIDE content filtered for at least five experiments (see Materials and Methods) there are no U-containing peptides.

It is evident that there is room for improvement in terms of MS repositories proteomics coverage of O- and U-containing peptides.

***Human UniProt UPI data set unicity contribution from human X-containing peptides***

- The X-containing peptides among the 257,506 (section 2 above) unique ones are 5,313 (2.1% of all the 257,506 ones; 31 from UniProtKB/Swiss-Prot and 5,282 from UniProtKB/TrEMBL). They come from 4,869 distinct sequences (5.8,% of all the 83,901 ones; of those 3 canonical and 3 isoform sequences from UniProtKB/Swiss-Prot and 5,307 sequences from UniProtKB/TrEMBL)

- The number of X residues per tryptic peptide ranges from 1 to 175

- Of these 4,869 sequences, 2,430 (49.9%; 3 canonical and 1 isoform sequences from UniProtKB/Swiss-Prot and 2,426 from UniProtKB/TrEMBL) have other unique peptides available, whereas 2,439 (50.1%; 2 isoform sequences from UniProtKB/Swiss-Prot and 2,437 sequences from UniProtKB/TrEMBL) only rely for their unicity on X-containing peptides and thus should not be considered as sequences containing unique peptides. These numbers point to the fact that contribution to unicity from X-containing peptides is low.

***Human UniProt UPI data set unicity contribution from human B-containing peptides***

- The B-containing peptides among the 257,506 (section 2 above) unique ones are 59 (0.02% of all the 257,506 ones; 38 from UniProtKB/Swiss-Prot and 21 from UniProtKB/TrEMBL). They come from 42 distinct sequences (0.05% of all the 83,901 ones; 22 canonical sequences from UniProtKB/Swiss-Prot and 20 sequences from UniProtKB/TrEMBL).

- The number of B residues per tryptic peptide ranges from 1 to 6.

- Of these 42 sequences, 36 (85.7%; 21 from UniProtKB/Swiss-Prot and 15 from UniProtKB/TrEMBL) have other unique peptides available, whereas 6 (14.3%; 1 from UniProtKB/Swiss-Prot and 5 from UniProtKB/TrEMBL) only rely for their unicity on B-containing peptides and thus should not be considered as sequences containing unique peptides. These numbers point to the fact that contribution to unicity from B-containing peptides is low.

***Human UniProt UPI data set unicity contribution from human Z-containing peptides***

- The Z-containing peptides among the 257,506 (section 2 above) unique ones are 40 (0.01% of all the 257,506 ones; 37 from UniProtKB/Swiss-Prot and 3 from UniProtKB/TrEMBL). They come from 29 distinct sequences (0.03% of all the 83,901 ones; 26 canonical sequences from UniProtKB/Swiss-Prot and 3 sequences from UniProtKB/TrEMBL).

- The number of Z residues per tryptic peptide ranges from 1 to 5.

- Of these 29 sequences, 27 (93.1%; 25 from UniProtKB/Swiss-Prot and 2 from UniProtKB/TrEMBL) have other unique peptides available, whereas 2 (6.9%; 1 from UniProtKB/Swiss-Prot and 1 from UniProtKB/TrEMBL) only rely for their unicity on Z-containing peptides and thus should not be considered as sequences containing unique peptides. These numbers point to the fact that contribution to unicity from Z-containing peptides is low.

***Human UniProt UPI data set unicity contribution from human peptides concurrently containing X, B and Z residues***

- No unique peptide contains at the same time X, B and Z residues.

- 5 unique peptides contain at the same time X and B residues.

- 2 unique peptides contain at the same time X and Z residues.

- 23 unique peptides contain at the same time B and Z residues.

- No entries have their unicity only based on peptides concurrently containing X, B and Z.

- 2,457 entries (2.9% of all the 83,901 ones; 2 canonical and 2 isoform sequences from UniProtKB/Swiss-Prot and 2,453 sequences from UniProtKB/TrEMBL) have their unicity based only on X- and/or B- and/or Z-containing peptides and thus should not be considered as sequences containing unique peptides.

***Ambiguous residues in MS proteomics repositories***

The MS proteomics repositories content in terms of these ambiguous residues is as follows.

1) GPMDB

- X-containing sequences for: *G. gallus* (1 sequence, 3 X residues), *H. sapiens* (9 sequences, 9 X residues), *M. musculus* (1 sequence, 1 X residue) and *R. norvegicus* (1 sequence, 1 X residue).

- No B- nor Z-containing sequences.

2) PeptideAtlas

- X-containing sequences for: *H. sapiens* (1 sequence, 1 X residue) and *S. cerevisiae* S288c (1 sequence, 1 X residue).

- B-containing sequences for: *H. sapiens* (7 sequences, 8 B residues).

- Z-containing sequences for: *H. sapiens* (6 sequences, 8 Z residues).

3) PRIDE

- X-containing sequences for: *A. thaliana* (5 sequences, 5 X residues), *B. taurus* (19 sequences, 31 X residues), *D. rerio* (122 sequences, 122 X residues), *D. melanogaster* (1,341 sequences, 2,053 X residues), *G. gallus* (76 sequences, 86 X residues), *H. sapiens* (1,384 sequences, 1,768 X residues), *H. sapiens* filtered for peptides found in at least five experiments (64 sequences, 80 X residues), *M. musculus* (1,352 sequences, 1,722 X residues), *M. musculus* filtered for peptides found in at least five experiments (72 sequences, 88 X residues), *R. norvegicus* (244 sequences, 254 X residues) and *S. cerevisiae* S288c (17 sequences, 17 X residues).

- B-containing sequences for: *D. melanogaster* (1 sequence, 1 B residue), *G. gallus* (1 sequence, 1 B residue), *H. sapiens* (42 sequences, 51 B residues), *H. sapiens* filtered for peptides found in at least five experiments (8 sequences, 10 B residues), *M. musculus* (6 sequences, 8 B residues), *R. norvegicus* (28 sequences, 49 B residues) and *S. cerevisiae* S288c (1 sequence, 1 B residue).

- Z-containing sequences for: *H. sapiens* (41 sequences, 57 Z residues), *H. sapiens* filtered for peptides found in at least five experiments (4 sequences, 8 Z residues), *M. musculus* (6 sequences, 12 Z residues) and *R. norvegicus* (25 sequences, 31 Z residues).

It must be noted that GPMDB and PeptideAtlas are likely reporting ambiguous residues containing sequences based on a sufficient amount of additional matches inside each of these sequences (as reported by search engines during their reprocessing) while PRIDE relies on original unfiltered submissions. Search engines are indeed capable of dealing directly with ambiguous peptides (if their presence in a sequence is not exceedingly high and if a sufficient amount of matched signals can be reached).

***UniProt complete proteomes (CPI data sets) vs. Ensembl***

From the comparisons reported in Table 3 and Supplementary Table 8 the following considerations can be extracted:

- The peptides unique to Ensembl data sets range from a value of 0.01% for *S. scrofa* to 1.1% for *D. melanogaster*. This indicates that only few peptides are missing from CPI data sets.

- The peptides unique to CPI data sets range from a value of 0.2% for *S. cerevisiae* to 2.8% for *H. sapiens*, indicating a higher information on average available in the CPI data sets.

- The shared peptides range from 96.7% for *H. sapiens* to 99.4% for *S. cerevisiae*, thus denoting a high average concordance between CPI and Ensembl data sets.

In terms of MS proteomics repositories info, the shared peptides which have experimental evidence range from 4.2% for *D. rerio* to 35.5% for *S. cerevisiae* with human displaying the highest number of shared peptides bearing an evidence (24.7% of the indicated ones). The percentages for non-shared peptides are not high either. All this points to a large space for improvements in terms of MS evidence coverage.

From the human and mouse comparisons reported in Supplementary Table 6 the following considerations can be done:

- Passing to the UniProt UPI data sets does not affect much neither the peptides unique to Ensembl nor the shared peptides while it affects the peptides unique to the UniProt data set in the comparison. This indicates an increase in sequence information in the UPI data sets which is not directly linked to the genome information as provided by Ensembl.

- The UPIV and UPID data sets follow this same trend: more sequence information is added to the UniProt data sets.

- From the other comparisons in Supplementary Table 6, the general sequence information carried by UniProtKB/Swiss-Prot isoforms and by UniProtKB/TrEMBL entries is evident as well the not so pronounced loss of information from the UniRef100 data sets.

With respect to MS proteomics repositories information, similar trends as the ones reported for Table 3 are found, so there is room for improvement in MS peptide coverage. The shared peptides from the UPIV and UPI data sets account for the highest coverage from MS evidence (the numbers are very similar to the ones for CPI data sets in Table 3 though) thus reflecting the fact that UPI, UPID and UPIV data sets contain more sequence information. Notably, in passing from CPI to UPI (same for UPID or UPIV) the number of MS evidence-bearing peptides unique to UniProt data sets increases consistently.

***UniProt complete proteomes (CPI data sets) vs. IPI***

From the comparisons reported in Table 3 and Supplementary Table 8 the following considerations can be extracted:

- The peptides unique to IPI data sets range from a value of 7.7% for *M. musculus* to 13.1% for *G. gallus*. The peptides unique to CPI data sets range from a value of 0.2% for *A. thaliana* to 4.6% for *B. taurus*. The shared peptides range from 82.7% for *B. taurus* to 91.4% for *M. musculus*. All this shows less concordance between the CPI and IPI data sets when compared to the CPI vs. Ensembl data sets. Nevertheless this is ameliorated by not limiting UniProt content to CPI data sets (see below).

In terms of MS proteomics repositories information, the shared peptides which have experimental evidence range from 4.4% for *D. rerio* to 24.7% for *H. sapiens*. The percentages for non-shared peptides are not high either. All this points to a large space for improvements in terms of MS evidence coverage.

From the human and mouse comparisons reported in Supplementary Table 6 the following considerations can be done:

- Passing to the UniProt UPI data sets does not affect much the shared peptides while it affects the peptides unique to the UniProt and the IPI data sets. This indicates an increase in sequence information in the UPI data sets. Indeed the human peptides unique to IPI go down to 3.9% (with respect to 10.1% for the CPI data set) while the mouse ones go down to 2.6% (with respect to 7.7% for the CPI data set). The poor evidence shared by the three MS proteomics repositories for these peptides, is evidenced in Supplementary Figure 1.

- The UPIV and UPID data sets follow this same trend: more sequence information is added to the UniProt data sets.

- From the other comparisons in Supplementary Table 6, the general sequence information carried by UniProtKB/Swiss-Prot isoforms and by UniProtKB/TrEMBL entries is evident as well as the not so pronounced loss of information from the UniRef100 data sets.

With respect to MS proteomics repositories information, similar trends as the ones reported for Table 3 are found, so there is room for improvement in MS peptide coverage. The shared peptides from the UPIV and UPI data sets account for the highest coverage from MS evidence (though the numbers are very similar to the ones for CPI data sets in Table 3) thus reflecting the fact that UPI, UPID and UPIV data sets contain more sequence information. As for the Ensembl comparisons, in passing from CPI to UPI (same for UPID or UPIV) the number of MS evidence-bearing peptides unique to UniProt data sets increase consistently.

***UniProt complete proteomes (CPI data sets) vs. RefSeq***

From the comparisons reported in Table 3 and Supplementary Table 8 the following considerations can be extracted:

- The peptides unique to RefSeq data sets range from a value of 0.01% for *S. cerevisiae* to 16.6% for *G. gallus*. The peptides unique to CPI data sets range from a value of 0.4% for *A. thaliana* to 15.6% for *D. rerio*. The shared peptides range from 70.8% for *S. scrofa* to 99.3% for *A. thaliana*. All this shows less average concordance between CPI and RefSeq data sets when compared to CPI vs. IPI data sets.

In terms of MS proteomics repositories info, the shared peptides which have experimental evidence range from 4.9% for *D. rerio* to 36.4% for *S. cerevisiae* with human displaying the highest number of shared peptides bearing an evidence (27.7% of the indicated ones). The percentages for non-shared peptides are not high either. All this points to a large space for improvements in terms of MS evidence coverage.

From the human and mouse comparisons reported in Supplementary Table 6 the following considerations can be extracted:

- Passing to the UniProt UPI data sets does not affect much the shared peptides while it affects the peptides unique to the UniProt and the RefSeq data sets. This indicates an increase in sequence information available in the UPI data sets.

- The UPIV and UPID data sets follow this same trend: more sequence information is added to the UniProt data sets.

- From the other comparisons in Supplementary Table 6, the general sequence information carried by UniProtKB/Swiss-Prot isoforms and by UniProtKB/TrEMBL entries is evident as well as the not so pronounced loss of information from the UniRef100 data sets.

With respect to MS proteomics repositories information, similar trends as the ones reported for Table 3 were found, so there is room for improvement in MS peptide coverage. In fact, the shared peptides from the UPIV and UPI data sets account for the highest coverage from MS evidence (though the numbers are very similar to the ones for CPI data sets in Table 3) thus reflecting the fact that UPI, UPID and UPIV data sets contain more sequence information. Notably, in passing from CPI to UPI (same for UPID or UPIV) the number of MS evidence-bearing peptides unique to UniProt data sets increases consistently but in a lesser degree when compared to with the comparisons between CPI and IPI data sets.

**Legends of the Supplementary Figures**

Supplementary Figure 1. UniProt vs. IPI.

Number of IPI tryptic peptides that do not have a corresponding one in the UniProtKB UPI data set, together with their evidence in the MS proteomics repositories. A-B: PRIDEF refers to tryptic peptides which are shared by at least five PRIDE experiments. The sum of the reported numbers for each graph is reported in Table 4. C-D: the above cited PRIDE filtering is not applied.

Supplementary Figure 2. Pairwise comparisons diagram.

Supplementary Figure 3. Sequence clustering and tryptic peptides.

Superimposed proteins A and B share the same aminoacidic sequence except for the sequence gap displayed by A. Residues open to tryptic cleavage are indicated. In light grey color is highlighted the peptide (divided by the gap in the graphics) which will be lost upon clustering the two sequences and merging them into a single entry with the sequence displayed in B. In the case where the A sequence is considered as two distinct sequences, the lost peptides will be the two light grey ones (the non-tryptic one on the left and the tryptic one on the right).

Supplementary Figure 4. All versus proteome sequences for UniProt.

For each organism two histogram bars are displayed. The bar on the left (tagged with the organism name) shows all the UniProtKB sequences, while the bar on the right (tagged with "Proteome") shows all the corresponding proteome UniProtKB sequences for that organism.

Iso: contribution from UniProtKB/Swiss-Prot isoform sequences. Can: contribution from UniProtKB/Swiss-Prot canonical sequences. TR: contribution from UniProtKB/TrEMBL sequences.

Supplementary Figure 1

15

5

30

90

11

66

23

PeptideAtlas

PRIDEF

gpmDB

**Human**

**A**

2

4

6

170

4

7

92

PeptideAtlas

PRIDEF

gpmDB

**Mouse**

**B**

29

8

16

1,753

29

48

20

PeptideAtlas

PRIDE

gpmDB

**Human**

**C**

5

29

3

2,279

7

4

67

PeptideAtlas

PRIDE

gpmDB

**Mouse**

**D**

Supplementary Figure 2

Peptides belonging only to DB1

DB1

*In silico* tryptic digest

*In silico* tryptic digest

DB2

Filter (e.g. short peptides)

Filter (e.g. short peptides)

Peptides belonging only to DB2

Peptides shared by DB1 and DB2

Pairwise comparison

Peptide occurrence in proteomics MS-repositories

I

II

III

**Supplementary Figure 3**

K or R

K or R

K or R

K or R

gap

A)

B)

**Supplementary Figure 4**

**Supplementary Tables**

Supplementary Table 1. Number of sequences in used databases.

Sections marked by double lines. Left section: sequence collections used in this work. Right sections: human and mouse UniProt additional sequence collections used in this work.

|  | **Ensembl** | **IPI** | **RefSeq** | **CPI** |  | **CPID** | **CP** | **CPIV** | **CPIR** | **CPIDR** |
| --- | --- | --- | --- | --- | --- | --- | --- | --- | --- | --- |
| ***A. thaliana*** |  | 39,677 | 35,375 | 33,317 | ***H. sapiens*** | 133,004 | 68,130 | 211,740 | 72,320 | 108,118 |
| ***B. taurus*** | 22,118 | 30,403 | 32,226 | 24,238 | ***M. musculus*** |  | 42,761 | 51,793 | 45,996 |  |
| ***C. elegans*** | 31,234 |  | 25,816 | 25,876 |  | **CPIVR** | **SPI** | **SPID** | **SP** | **SPIV** |
| ***C. l. familiaris*** | 25,160 |  | 21,985 | 26,474 | ***H. sapiens*** | 182,217 | 36,991 | 85,107 | 20,233 | 163,843 |
| ***D. rerio*** | 42,166 | 40,470 | 27,269 | 40,575 | ***M. musculus*** | 45,056 | 24,421 |  | 16,566 | 25,598 |
| ***D. melanogaster*** | 23,849 |  | 24,122 | 18,797 |  | **UPI** | **UPID** | **UP** | **UPIV** | **UPIR** |
| ***G. gallus*** | 22,194 | 25,992 | 17,711 | 23,625 | ***H. sapiens*** | 148,042 | 196,158 | 131,284 | 274,894 | 106,584 |
| ***H. sapiens*** | 97,348 | 91,464 | 34,677 | 84,888 | ***M. musculus*** | 82,667 |  | 74,812 | 83,844 | 63,182 |
| ***M. musculus*** | 50,877 | 59,534 | 30,164 | 50,616 |  | **UPIDR** | **UPIVR** | **TR** |  |  |
| ***R. norvegicus*** | 32,971 | 39,925 | 29,739 | 37,175 | ***H. sapiens*** | 149,046 | 222,698 | 111,051 |  |  |
| ***S. cerevisiae* S288c** | 6,692 |  | 5,907 | 6,651 | ***M. musculus*** |  | 64,057 | 58,246 |  |  |
| ***S. scrofa*** | 23,118 |  | 24,571 | 23,724 |  |  |  |  |  |  |

Supplementary Table 2 Details of the protein data sets used in this study (no human and no mouse).

| **Database** | **Species** | **Release** | **Set specification** | | | | | **Abbreviation** |
| --- | --- | --- | --- | --- | --- | --- | --- | --- |
| UniProtKB | *A. thaliana*  *B. taurus*  *C. elegans*  *C. l. familiaris*  *D. rerio*  *D. melanogaster*  *G. gallus*  *R. norvegicus*  *S. cerevisiae* S288c  *S. scrofa* | 2012_10 | UniProtKB complete proteome set sequences (UniProtKB/Swiss-Prot canonical and isoforms plus UniProtKB/TrEMBL, all with KW-0181). The keyword KW-0181 refers to complete proteomes (www.uniprot.org/docs/keywlist) | | | | | CPI |
|  | | | | | | | | |
| **Database** | **Species** | **Release** | **Database** | **Species** | **Release** | **Database** | **Species** | **Release** |
| RefSeq | *A. thaliana*  *B. taurus*  *C. elegans*  *C. l. familiaris*  *D. rerio*  *D. melanogaster*  *G. gallus*  *R. norvegicus*  *S. cerevisiae* S288c  *S. scrofa* | 55 | Ensembl | *B. taurus*  *C. elegans*  *C. l. familiaris*  *D. rerio*  *D. melanogaster*  *G. gallus*  *R. norvegicus*  *S. cerevisiae* S288c  *S. scrofa* | 68 | IPI | *A. thaliana*  *B. taurus*  *D. rerio*  *G. gallus*  *R. norvegicus* | 09/2011 |

Supplementary Table 3. Number of peptides in MS proteomics repositories.

Numbers of peptides retrieved from MS proteomics repositories. Only peptides with a length of six or more AAs were retrieved. In brackets: the number on the left is the percentage of peptides having K or R at their C-terminus while the number on the right represents the percentage of peptides, among those having K or R at their C-terminus, containing one or more tryptic missed-cleavage sites. * Human and mouse PRIDE sequences filtered to be present in at least five experiments. When more than one MS proteomics repository has content for one of the species, the "Total" column reports the summed numbers where identical sequences are counted as one.

|  | **PRIDE** | **GPMDB** | **PeptideAtlas** | **Total** |
| --- | --- | --- | --- | --- |
| ***A. thaliana*** | 266,987 (96.0, 37.0) | 123,814 (91.9, 21.4) |  | 294,152 (94.8, 36.3) |
| ***B. taurus*** | 21,111 (80.7, 38.3) | 140,062 (87.7, 26.3) | 8,559 (97.6, 13.8) | 158,628 (86.6, 27.9) |
| ***C. elegans*** | 113,105 (94.6, 32.8) | 88,622 (92.09, 29.0) | 75,647 (97.7, 33.3) | 142,127 (92.5, 33.1) |
| ***C. l. familiaris*** |  | 141,534 (88.0, 26.5) |  |  |
| ***D. rerio*** | 32,237 (97.2, 39.9) | 46,545 (88.2, 24.9) |  | 66,046 (90.9, 31.6) |
| ***D. melanogaster*** | 221,932 (98.2, 76.4) | 106,331 (89.2, 25.9) | 58,746 (91.7, 25.0) | 326,664 (94.5, 60.3) |
| ***G. gallus*** | 21,830 (98.4, 51.0) | 68,926 (89.2, 25.9) |  | 86,273 (91.1, 32.4) |
| ***H. sapiens*** | 137,726* (97.3, 37.4) | 270,541 (86.0, 26.5) | 239,609 (85.1, 23.8) | 435,724 (84.4, 30.3) |
| ***H. sapiens*** (no filtering) | 845,984 (93.6, 54.3) |  |  |  |
| ***M. musculus*** | 73,924* (97.03, 39.1) | 194,843 (88.2, 24.8) | 37,494 (94.6, 16.0) | 241,139 (89.5, 29.5) |
| ***M. musculus*** (no filtering) | 722,929 (94.16, 58.5) |  |  |  |
| ***R. norvegicus*** | 203,678 (98.40, 46.7) | 217,697 (87.8, 26.3) |  | 376,353 (92.3, 37.9) |
| ***S. cerevisiae* S288c** | 34,180 (94.68, 24.9) | 155,445 (73.1, 22.2) | 59,279 (94.5, 25.7) | 174,652 (75.2, 24.0) |
| ***S. scrofa*** |  | 105,385 (87.6, 26.5) | 138,83 (97.6, 18.9) | 112,985 (88.2, 26.0) |

Supplementary Table 4. UniProtKB 2012_10 isoform and variant statistics.

Isoform and variant statistics for the organisms used in this work in terms of number of sequences and percentage.

|  | **Isoform sequences** | | **Sequences with variants** | |
| --- | --- | --- | --- | --- |
| ***Total*** | *32,585* | *%* | *16,708* | *%* |
| ***A. thaliana*** | 1,409 | 4.3 | 130 | 0.8 |
| ***B. taurus*** | 363 | 1.1 | 107 | 0.6 |
| ***C. elegans*** | 876 | 2.7 | 16 | 0.1 |
| ***C. l. familiaris*** | 44 | 0.1 | 38 | 0.2 |
| ***D. rerio*** | 204 | 0.6 | 5 | 0.03 |
| ***D. melanogaster*** | 1,264 | 3.9 | 238 | 1.4 |
| ***G. gallus*** | 230 | 0.7 | 42 | 0.2 |
| ***H. sapiens*** | 16,758 | 51.4 | 12,456 | 74.6 |
| ***M. musculus*** | 7,855 | 24.1 | 365 | 2.2 |
| ***R. norvegicus*** | 1,525 | 4.7 | 115 | 0.7 |
| ***S. cerevisiae* S288c** | 22 | 0.1 | 131 | 0.8 |
| ***S. scrofa*** | 70 | 0.2 | 55 | 0.3 |

Supplementary Table 5 Peptide unicity table for the various data sets/organisms.

For each organism and each data set (abbreviations are reported in the main text), the total number of tryptic peptides is reported together with the percentage of unique peptides in brackets.

|  | **Ensembl** | **IPI** | **RefSeq** | **CPI** |
| --- | --- | --- | --- | --- |
| ***A. thaliana*** |  | 670,411 (71.0) | 607,105 (73.9) | 608,531 (78.3) |
| ***B. taurus*** | 579,981 (88.8) | 639,240 (69.3) | 612,337 (61.4) | 585,536 (81.9) |
| ***C. elegans*** | 462,499 (64.7) |  | 461,080 (77.1) | 462,568 (77.0) |
| ***C. l. familiaris*** | 599,136 (70.3) |  | 586,496 (87.0) | 602,755 (66.3) |
| ***D. rerio*** | 711,954 (59.0) | 775,574 (74.4) | 685,695 (90.8) | 715,410 (62.2) |
| ***D. melanogaster*** | 408,559 (61.6) |  | 408,676 (60.8) | 407,040 (73.8) |
| ***G. gallus*** | 456,374 (74.7) | 514,667 (74.4) | 488,786 (93.4) | 464,661 (71.8) |
| ***H. sapiens*** | 680,200 (33.4) | 759,094 (40.4) | 609,863 (61.7) | 696,041 (38.1) |
| ***M. musculus*** | 640,354 (53.5) | 697,473 (48.3) | 618,868 (75.7) | 650,422 (50.4) |
| ***R. norvegicus*** | 603,491 (64.5) | 673,887 (58.5) | 613,908 (69.0) | 622,094 (58.1) |
| ***S. cerevisiae* S288c** | 164,320 (97.7) |  | 159,629 (97.7) | 163,987 (97.4) |
| ***S. scrofa*** | 502,742 (84.2) |  | 529,335 (84.4) | 507,497 (82.4) |

Supplementary Table 6. Additional human and mouse tryptic search spaces pairwise comparisons between UniProt data sets and those from other sequence providers.

Each pairwise comparison is squared off by double lines and the corresponding two data sets (DB) are indicated next to the numbers of peptides unique to each of them. "Peptides" indicates the number of tryptic peptides for each of the three compartments of the comparisons (I, II and III in Supplementary Figure 2). "Com." indicates the number of tryptic peptides shared by both data sets in each pairwise comparison. Corresponding percentages of peptides which are found in MS proteomics repositories are reported in brackets for each of the three compartments of the comparisons. Mouse comparisons are highlighted with a light grey background.

| **DB** | **Peptides** | **DB** | **Peptides** | **DB** | **Peptides** | **DB** | **Peptides** | **DB** | **Peptides** |
| --- | --- | --- | --- | --- | --- | --- | --- | --- | --- |
| Esembl | 3,636 (5.6) | Esembl | 16,646 (4.5) | Esembl | 3,470 (5.4) | Esembl | 77,262 (1.2) | Esembl | 77,249 (1.2) |
| CPID | 41,997 (2.2) | CP | 13,475 (5.7) | CPIV | 80,785 (1.7) | SPI | 19,419 (4.2) | SPID | 41,937 (2.2) |
| Com. | 676,564 (24.7) | Com. | 663,554 (25.1) | Com. | 676,730 (24.7) | Com. | 602,938 (27.6) | Com. | 602,951 (27.6) |
| Esembl | 97,229 (2.2) | Esembl | 76,738 (1.1) | Esembl | 2,603 (3.0) | Esembl | 13,573 (3.2) | Esembl | 2,492 (3.0) |
| SP | 13,414 (5.7) | SPIV | 80,725 (1.7) | UPID | 126,235 (1.5) | UP | 98,313 (1.8) | UPIV | 161,373 (1.3) |
| Com. | 582,971 (28.4) | Com. | 603,462 (27.6) | Com. | 677,597 (24.7) | Com. | 666,627 (25.1) | Com. | 677,708 (24.7) |
| Esembl | 12,469 (1.7) | Esembl | 11,144 (1.8) | Esembl | 10,901 (1.7) |  |  |  |  |
| UPIR | 97,520 (1.7) | UPIDR | 120,953 (1.4) | UPIVR | 155,923 (1.3) |  |  |  |  |
| Com. | 667,731 (25.1) | Com. | 669,056 (25.0) | Com. | 669,299 (25.0) |  |  |  |  |
| IPI | 78,464 (1.1) | IPI | 96,840 (1.5) | IPI | 77,467 (1.0) | IPI | 138,184 (1.1) | IPI | 138,149 (1.1) |
| CPID | 37,931 (0.6) | CP | 14,775 (0.9) | CPIV | 75,888 (0.8) | SPI | 1,447 (5.2) | SPID | 23,943 (0.6) |
| Com. | 680,630 (24.7) | Com. | 662,254 (25.3) | Com. | 681,627 (24.7) | Com. | 620,910 (27.0) | Com. | 620,945 (27.0) |
| IPI | 163,450 (1.7) | IPI | 136,837 (1.0) | IPI | 31,885 (0.8) | IPI | 47,819 (1.3) | IPI | 31,699 (0.7) |
| SP | 741 (8.8) | SPIV | 61,930 (0.9) | UPID | 76,623 (0.9) | UP | 53,665 (1.1) | UPIV | 111,686 (0.8) |
| Com. | 595,644 (27.9) | Com. | 622,257 (26.9) | Com. | 727,209 (23.2) | Com. | 711,275 (23.7) | Com. | 727,395 (23.2) |
| IPI | 40,775 (0.9) | IPI | 38,828 (0.9) | IPI | 38,555 (0.9) |  |  |  |  |
| UPIR | 46,932 (1.0) | UPIDR | 69,743 (0.8) | UPIVR | 104,683 (0.7) |  |  |  |  |
| Com. | 718,319 (23.5) | Com. | 720,266 (23.4) | Com. | 720,539 (23.4) |  |  |  |  |
| RefSeq | 9,023 (1.7) | RefSeq | 17,182 (3.7) | RefSeq | 8,440 (0.9) | RefSeq | 16,254 (2.5) | RefSeq | 16,253 (2.5) |
| CPID | 117,721 (1.5) | CP | 84,348 (1.8) | CPIV | 156,092 (1.4) | SPI | 28,748 (4.0) | SPID | 51,278 (2.4) |
| Com. | 600,840 (27.7) | Com. | 592,681 (28.0) | Com. | 601,423 (27.7) | Com. | 593,609 (28.0) | Com. | 593,610 (28.0) |
| RefSeq | 28,645 (4.9) | RefSeq | 15,498 (1.8) | RefSeq | 6,823 (0.8) | RefSeq | 13,279 (2.7) | RefSeq | 6,493 (0.6) |
| SP | 15,167 (6.2) | SPIV | 89,822 (1.9) | UPID | 200,792 (1.3) | UP | 168,356 (1.5) | UPIV | 235,711 (1.2) |
| Com. | 581,218 (28.4) | Com. | 594,365 (28.0) | Com. | 603,040 (27.6) | Com. | 596,584 (27.9) | Com. | 603,370 (27.6) |
| RefSeq | 8,138 (1.1) | RefSeq | 7,374 (1.1) | RefSeq | 6,983 (0.8) |  |  |  |  |
| UPIR | 163,526 (1.5) | UPIDR | 187,520 (1.3) | UPIVR | 222,342 (1.2) |  |  |  |  |
| Com. | 601,725 (27.7) | Com. | 602,489 (27.6) | Com. | 602,880 (27.6) |  |  |  |  |
| Esembl | 7,366 (6.8) | Esembl | 3,045 (7.6) | Esembl | 139,471 (6.3) | Esembl | 146,076 (6.4) | Esembl | 139,345 (6.3) |
| CP | 8,212 (5.6) | CPIV | 13,967 (4.2) | SPI | 13,128 (4.5) | SP | 8,200 (5.6) | SPIV | 13,955 (4.2) |
| Com. | 632,988 (17.3) | Com. | 637,309 (17.2) | Com. | 500,883 (20.2) | Com. | 494,278 (20.3) | Com. | 501,009 (20.2) |
| Esembl | 5,606 (5.4) | Esembl | 2,173 (5.8) | Esembl | 6,450 (3.7) | Esembl | 5,469 (3.8) |  |  |
| UP | 65,733 (2.4) | UPIV | 71,020 (2.3) | UPIR | 65,312 (2.4) | UPIVR | 66,625 (2.4) |  |  |
| Com. | 634,748 (17.2) | Com. | 638,181 (17.2) | Com. | 633,904 (17.3) | Com. | 634,885 (17.3) |  |  |
| IPI | 62,834 (2.7) | IPI | 53,929 (2.5) | IPI | 184,983 (5.3) | IPI | 196,119 (5.3) | IPI | 184,820 (5.3) |
| CP | 6,561 (1.8) | CPIV | 7,732 (1.6) | SPI | 1,521 (3.2) | SP | 1,124 (4.2) | SPIV | 2,311 (2.4) |
| Com. | 634,639 (17.3) | Com. | 643,544 (17.1) | Com. | 512,490 (19.8) | Com. | 501,354 (20.1) | Com. | 512,653 (19.8) |
| IPI | 29,635 (1.9) | IPI | 18,967 (1.5) | IPI | 23,657 (1.8) | IPI | 22,388 (1.7) |  |  |
| UP | 26,627 (0.9) | UPIV | 30,695 (0.8) | UPIR | 25,400 (0.8) | UPIVR | 26,425 (0.8) |  |  |
| Com. | 670,846 (16.5) | Com. | 678,506 (16.4) | Com. | 673,816 (16.5) | Com. | 675,085 (16.4) |  |  |
| RefSeq | 22,547 (2.1) | RefSeq | 19,884 (1.4) | RefSeq | 121,254 (6.6) | RefSeq | 125,313 (6.7) | RefSeq | 121,116 (6.6) |
| CP | 44,879 (3.3) | CPIV | 52,292 (3.2) | SPI | 16,397 (5.4) | SP | 8,923 (7.0) | SPIV | 17,212 (5.1) |
| Com. | 596,321 (18.2) | Com. | 598,984 (18.1) | Com. | 497,614 (20.2) | Com. | 493,555 (20.3) | Com. | 497,752 (20.2) |
| RefSeq | 18,217 (1.5) | RefSeq | 16,242 (1.0) | RefSeq | 17,245 (1.2) | RefSeq | 16,550 (1.1) |  |  |
| UP | 99,830 (2.6) | UPIV | 106,575 (2.6) | UPIR | 97,593 (2.7) | UPIVR | 99,192 (2.6) |  |  |
| Com. | 600,651 (18.1) | Com. | 602,626 (18.0) | Com. | 601,623 (18.0) | Com. | 602,318 (18.0) |  |  |

Supplementary Table 7. UniProtKB 2012_10 statistics (all species).

Sections marked by double lines. Upper section: global UniProt statistics in terms of number of sequences with the detail for the two UniProt sections (UniProtKB/Swiss-Prot isoforms not included). Lower section: corresponding aminoacidic frequencies. In square brackets the frequencies for the 32,585 UniProtKB/Swiss-Prot isoform sequences. Total figures for frequencies which include UniProtKB/Swiss-Prot isoforms are the sum of the two numbers inside and outside the brackets.

| **UniProtKB entries** | | | **27,661,073** |
| --- | --- | --- | --- |
| UniProtKB/Swiss-Prot section, canonical sequences | | | 538,259 |
| UniProtKB/TrEMBL section | | | 27,122,814 |
|  | | | |
| **AA** | **Frequency** | **AA** | **Frequency** |
| A | 772,229,283 [1,364,393] | N | 368,434,683 [749,062] |
| B | 21,533 [2] | O | 45 [0] |
| C | 112,554,561 [402,110] | P | 419,684,803 [1,231,863] |
| D | 476,621,002 [988,145] | Q | 354,580,300 [964,242] |
| E | 555,651,017 [1,436,990] | R | 486,851,706 [1,107,767] |
| F | 360,342,593 [687,801] | S | 596,060,958 [1,685,408] |
| G | 634,677,103 [1,275,388] | T | 499,099,430 [1,095,593] |
| H | 198,128,670 [493,758] | U | 1,935 [33] |
| I | 537,514,766 [881,876] | V | 607,143,106 [1,200,169] |
| J | 0 [0] | W | 115,877,457 [227,552] |
| K | 475,850,392 [1150141] | X | 3,321,540 [156] |
| L | 887,607,215 [1887627] | Y | 273,109,738 [516398] |
| M | 221,032,355 [432,037] | Z | 7,734 [0] |

Supplementary Table 8. Pairwise comparisons of UniProt data sets tryptic search spaces vs. other data sets.

Each pairwise comparison is squared off by double lines and the corresponding two data sets (DB) are indicated next to the numbers of peptides unique to each of them. "Peptides" indicates the number of tryptic peptides for each of the three compartments of the comparisons (I, II and III in Supplementary Figure 2). "Com." indicates the number of tryptic peptides shared by both data sets in each pairwise comparison. Corresponding percentages of peptides which are found in MS proteomics repositories are reported in brackets for each of the three compartments of the comparisons.

| **Organism** | **DB** | **Peptides** | **DB** | **Peptides** | **DB** | **Peptides** |
| --- | --- | --- | --- | --- | --- | --- |
| ***A. thaliana*** |  |  | IPI | 63,570 (0.4) | RefSeq | 1,317 (5.0) |
|  |  |  | CPI | 1,690 (12.4) | CPI | 2,743 (0.7) |
|  |  |  | Com. | 606,841 (20.2) | Com. | 605,788 (20.2) |
| ***B. taurus*** | Ensembl | 166 (10.8) | IPI | 84,893 (1.3) | RefSeq | 81,015 (1.2) |
|  | CPI | 5,721 (6.9) | CPI | 31,189 (1.5) | CPI | 54,214 (2.3) |
|  | Com. | 579,815 (11.6) | Com. | 554,347 (12.1) | Com. | 531,322 (12.5) |
| ***C. elegans*** | Ensembl | 1,869 (2.0) |  |  | RefSeq | 983 (3.4) |
|  | CPI | 1,938 (0.9) |  |  | CPI | 2,471 (13.2) |
|  | Com. | 460,630 (14.9) |  |  | Com. | 460,097 (14.9) |
| ***C. l. familiaris*** | Ensembl | 467 (18.2) |  |  | RefSeq | 58,312 (1.7) |
|  | CPI | 4,086 (2.4) |  |  | CPI | 74,571 (1.0) |
|  | Com. | 598,669 (10.4) |  |  | Com. | 528,184 (11.7) |
| ***D. rerio*** | Ensembl | 904 (1.6) | IPI | 89,615 (0.6) | RefSeq | 97,322 (0.6) |
|  | CPI | 4,360 (1.8) | CPI | 29,451 (0.5) | CPI | 127,037 (0.8) |
|  | Com. | 711,050 (4.2) | Com. | 685,959 (4.4) | Com. | 588,373 (4.9) |
| ***D. melanogaster*** | Ensembl | 4,717 (1.6) |  |  | RefSeq | 4,993 (1.6) |
|  | CPI | 3,198 (6.3) |  |  | CPI | 3,357 (6.4) |
|  | Com. | 403,842 (19.9) |  |  | Com. | 403,683 (19.9) |
| ***G. gallus*** | Ensembl | 64 (1.5) | IPI | 69,844 (0.7) | RefSeq | 92,379 (0.6) |
|  | CPI | 8,351 (3.1) | CPI | 19,838 (1.8) | CPI | 68,254 (1.8) |
|  | Com. | 456,310 (7.8) | Com. | 444,823 (8.0) | Com. | 396,407 (8.7) |
| ***R. norvegicus*** | Ensembl | 330 (11.5) | IPI | 61,835 (3.1) | RefSeq | 72,023 (2.9) |
|  | CPI | 18,933 (13.1) | CPI | 10,042 (5.1) | CPI | 80,209 (5.7) |
|  | Com. | 603,161 (19.9) | Com. | 612,052 (19.9) | Com. | 541,885 (21.7) |
| ***S. cerevisiae* S288c** | Ensembl | 702 (0.3) |  |  | RefSeq | 17 (0) |
|  | CPI | 369 (6.2) |  |  | CPI | 4,375 (1.0) |
|  | Com. | 163,618 (35.5) |  |  | Com. | 159,612 (36.4) |
| ***S. scrofa*** | Ensembl | 56 (1.8) |  |  | RefSeq | 99,687 (2.3) |
|  | CPI | 4,811 (9.2) |  |  | CPI | 77,849 (2.2) |
|  | Com. | 502,686 (9.6) |  |  | Com. | 429,648 (11.0) |
